# Uterine serous carcinoma: assessing association between genomics and patterns of metastasis

**DOI:** 10.3389/fonc.2023.1066427

**Published:** 2023-05-09

**Authors:** Francesco Alessandrino, Nicole Goncalves, Sarah Wishnek Metalonis, Cibele Luna, Matthew M. Mason, Jiangnan Lyu, Marilyn Huang

**Affiliations:** ^1^ Department of Radiology, University of Miami, Miami, FL, United States; ^2^ University of Miami Miller School of Medicine, Miami, FL, United States; ^3^ Division of Biostatistics, Department of Public Health Science, University of Miami, Miami, FL, United States; ^4^ Division of Gynecologic Oncology, Sylvester Comprehensive Cancer Center, University of Miami, FL, United States

**Keywords:** uterine serous cancer, next generation sequencing, endometrial cancer, somatic mutations, recurrence, metastases

## Abstract

**Background:**

Uterine serous carcinoma (USC) is an aggressive subtype of endometrial carcinoma which has been increasing at alarming rates, particularly among Asian, Hispanic and Black women. USC has not been well characterized in terms of mutational status, pattern of metastases and survival.

**Objective:**

To investigate the association between sites of recurrence and metastases of USC, mutational status, race, and overall survival (OS).

**Methods:**

This single-center retrospective study evaluated patients with biopsy-proven USC that underwent genomic testing between January 2015 and July 2021. Association between genomic profile and sites of metastases or recurrence was performed using χ2 or Fisher’s exact test. Survival curves for ethnicity and race, mutations, sites of metastasis/recurrence were estimated using the Kaplan-Meier method and compared with log-rank test. Cox proportional hazard regression models were used to examine the association between OS with age, race, ethnicity, mutational status, and sites of metastasis/recurrence. Statistical analyses were performed using SAS Software Version 9.4.

**Results:**

The study included 67 women (mean age 65.8 years, range 44-82) with 52 non-Hispanic women (78%) and 33 Black women (49%). The most common mutation was *TP53* (55/58 women, 95%). The peritoneum was the most common site of metastasis (29/33, 88%) and recurrence (8/27, 30%). PR expression was more common in women with nodal metastases (p=0.02) and non-Hispanic women (p=0.01). *ERBB2* alterations were more common in women with vaginal cuff recurrence (p=0.02), while *PIK3CA* mutation was more common in women with liver metastases (p=0.048). *ARID1A* mutation and presence of recurrence or metastases to the liver were associated with lower OS (Hazard Ratio (HR): 31.87; 95%CI: 3.21, 316.9; p<0.001 and HR: 5.66; 95%CI: 1.2, 26.79; p=0.01, respectively). In the bivariable Cox model, the presence of metastasis/recurrence to the liver and/or the peritoneum were both independent significant predictors of OS (HR: 9.8; 95%CI: 1.85-52.7; p=0.007 and HR: 2.7; 95%CI: 1.02-7.1; p=0.04, respectively).

**Conclusions:**

*TP53* is often mutated in USC, which most commonly metastasize and recur in the peritoneum. OS was shorter in women with *ARID1A* mutations and with metastasis/recurrence to the liver. The presence of metastasis/recurrence to liver and/or peritoneum were independently associated with shorter OS.

## Introduction

Endometrial cancer (EC) is the most common gynecologic malignancy in the United States (1, 2) and is increasing at an alarming rate. Based on clinical and histological variables, EC have been divided into two types. Type I tumors, or endometrioid EC, represent approximately 85% of cases, and are usually low-grade with favorable outcomes. Type II tumors, or non-endometrioid EC, typically arise in postmenopausal patients, and are frequently of high-grade thus contributing to a relatively poor prognosis (3, 4). Type II EC is largely comprised of uterine serous carcinomas (USC), which represents less than 10% of all endometrial cancers, yet accounts for more than half of deaths attributed to EC (4–6). While type I are estrogen-dependent, the role of estrogens in type II EC is less clear, although studies have shown that the pathogenesis of type II EC may at least partially depend on the level of estrogens, differently from what was previously believed (7).

Pathological reporting of EC has limitations due to poor reproducibility of tumor typing and to accurately identify patients at risk for recurrence or metastatic disease (8). The identification of the underlying molecular background of EC has resulted in the development of new molecular based classifications of EC, namely The Cancer Genome Atlas (TCGA), which stratifies EC into four distinct clusters with prognostic significance: polymerase ε (POLE) ultramutated, copy-number low, and microsatellite instability (MSI) hypermutated, and copy-number high (9, 10).

The genomic characterization of USC is distinct from endometrioid EC, with USC exhibiting a high frequency of genetic aberrations involving *TP53*, *FBXW7*, *PPP2R1A* and *ARID1A.*USC are mostly classified as copy-number high according to TCGA (10, 11). In contrast, endometrioid EC typically demonstrates a higher frequency of microsatellite instability, frequent activation of WNT/CTNNB1 signaling, and mutations of *POLE*, *KRAS*, and *CTNNB1*. Endometrioid EC are mostly classified as copy-number low according to TCGA (9–11).

Although EC is typically diagnosed early and associated with a high 5-year survival of 80-90%, USCs have higher recurrence rates and carry a poor prognosis with significantly lower survival (4, 12). USCs have high risk of recurrence (up to 80%) and are associated with an increased incidence of extrauterine disease on presentation (5, 12–15). Hence, the ability to reduce mortality of EC largely depends on developing tailored therapy and management for recurrent and advanced USC (16).

Patterns of recurrence and metastasis may provide prognostic information for EC. For example, patients with single-site local or nodal recurrence of EC have been associated with improved survival compared to those with pelvic recurrence or distant metastasis (17, 18), while patients with peritoneal carcinomatosis or multiple sites of recurrence have significantly worse post-relapse survival rates (18).

Comprehensive genomic analysis of USC provides a clearer understanding of the molecular pathways involved in oncogenesis (19). Knowledge of the somatic mutations may help predict patterns of metastases and recurrence in various cancers, including urothelial cancer, where patients with *TP53* mutations are at a higher risk of lymph nodes metastases (12, 20–22).

The primary objective of this study is to investigate the association of somatic mutations occurring in a diverse patient population diagnosed with USC to the patterns of metastases, recurrence, overall survival (OS) and recurrence free survival (RFS).

## Materials and methods

### Patients and histopathologic data

This institutional review board (IRB)-approved, Health Insurance Portability and Accountability Act (HIPAA)-compliant retrospective study was performed at a single institution on consecutive patients diagnosed with USC who underwent somatic molecular testing between January 2015 and July 2021. Patients were excluded for 1) non-serous or mixed histology, or 2) data on recurrence or metastasis was not available in the electronic medical records (EMR).

### Clinical and histopathologic data

Medical records in the EMR were reviewed to extract clinicopathologic data of the patients by (C.L.) (M.M.) (N.G.) (M.H.). The collected information included demographics; date and stage at diagnosis; histopathology and genomic testing results; initial treatment (systemic therapy, radiotherapy, or surgery); date and sites of metastatic and recurrent disease; treatment modality for recurrent disease; progression date; and date of death or last follow-up.

### Imaging review

Cross-sectional images (Computed tomography (CT) of the chest, abdomen, and pelvis; FDG- Positron emission tomography (PET)/CT, and Magnetic resonance Imaging (MRI)) and reports were reviewed initially by a cancer imaging fellow (C.L.) and separately by a fellowship-trained cancer imaging radiologist with 5 years of experience (F.A.). Discrepancies were resolved by consensus between the two radiologists. The date of the first imaging showing metastasis or recurrence was recorded. Reports and images, when available, were analyzed to record sites of metastatic or recurrent disease, including pelvis, lung, liver, pleura, lymph nodes, peritoneum, bones, brain, and muscle for all patients. Lymph node involvement was determined by short-axis diameter ≥1.0 cm. Any new lesion identified at follow-up imaging after curative treatment was defined as recurrence based on pathologic confirmation whenever possible, or if it showed unequivocal growth on follow-up imaging, defined as >20% increase in the sum of diameters compared to baseline or nadir (with an absolute increase of at least 5 mm) according to RECIST 1.1 (23). Any extrauterine lesion present before curative treatment was performed was considered metastatic, based on pathologic confirmation whenever possible, or if it showed unequivocal growth on follow-up imaging exams according to RECIST 1.1 (23).

### Molecular testing

Molecular profiling was performed with immunohistochemistry (IHC) and next-generation sequencing (NGS) on either the primary tissue at diagnosis or from tissue obtained at recurrence. A board-certified gynecologic oncologist (M.H.) assigned a TCGA cluster based on the mutational profile (10). IHC and molecular testing was performed using either Caris Life Sciences (Caris Life Sciences, Phoenix, AZ, USA) or FoundationOne CDx (Foundation medicine inc., Cambridge, MA, USA) genomic profiling assays.

The IHC assays were performed using FDA-approved companion diagnostic or FDA-cleared tests consistent with the manufacturer’s instructions: ALK (Ventana ALK (D5F3) CDxAssay; ER (confirm anti-estrogen receptor ER, SP1, Ventana; FOLR1 (Ventana FOLR1-2.1 RxDx, Ventana; PR (confirm anti-progesterone PR (1E2), Ventana); HER2/neu (pathway anti-HER-2/neu (4B5), Ventana; PD-L1 22c3. pharmDx, Dako; Mismatch repair (MMR) proteins (MLH1, MSH2, MSH6, and PMS2; Ventana MMR RxDx Panel, Ventana). For ER/PR, staining intensity was classified as 0, 1+, 2+, 3+. The intensity thresholds for a positive test for ER were >/= 2+ with >/= 75% or >/=3+ with >/=50% of cells stained, for PR were >/=1+ and >/= 10% of cells stained.

Regarding molecular testing, details of specific NGS testing are available from Caris Life Sciences or FoundationOne CDx (24, 25). Among the genes covered, we focused our mutational analysis on the 12 genes most frequently mutated in this cohort.

### Statistical analysis

Categorical variables were summarized using frequencies and percentages and the continuous variable, age, was summarized using mean and standard deviation. Different genetic aberrations involving the same gene were grouped under that gene. The association of mutational status with the location of metastases or recurrence was assessed using Chi-square tests or Fisher’s exact tests when 20% or more of the frequency cells had expected counts less than 5.

OS was measured from the date of initial diagnosis of USC to death from any cause, censored at the date of the last follow-up in alive patients. Stratified Kaplan-Meier survival curves were generated, and univariable Cox proportional hazards regression was used to examine the association of ethnicity, race, mutational status, sites of recurrence or metastases with OS. OS curves were computed in all patients. Statistical significance was determined through the log-rank test. A p-value <0.05 was considered statistically significant.

Multivariable Cox proportional hazard regression with backward stepwise selection was performed with OS as response. The variables that had log-rank p-values<0.20 and sample size > 50 were used as the predictors in the model. Likelihood Ratio tests were used to test model predictability and test significance for additional individual and/or collection of variables. Hazards ratios and 95% CIs were calculated in each model to determine association and significant predictors of survival. Statistical computations were performed, and output was generated using SAS Software Version 9.4 (The SAS Institute, Cary, NC).

## Results

### Patient characteristics

From a total of 134 patients with EC who underwent genomic profiling, 67 patients with USC were included in the final study analysis. Fifty-eight patients with endometrioid histology, 5 patients with clear cell histology, 3 patients with mixed histology, and 1 patient with no data on recurrence or metastasis were excluded. Characteristics of the included subjects are summarized in **Table 1**. The average age was 65.8 years (Standard Deviation=8.3 years). Fifty-two patients were non-Hispanic (78%), 33 patients were Black (49%), 32 were White (48%), 1 was Asian (1.5%), and 1 patient self-reported multi-racial (1.5%). Thirty-three patients had evidence of metastases at time of diagnosis (49.2%), 27 patients had evidence of recurrence during follow-up (40.3%), and 7 patients did not show evidence of recurrence or metastasis during follow-up (10.5%). Median follow up was 21 months (range 20 days-10.2 years).

**Table 1:** Clinical characteristics of 67 patients with uterine serous carcinoma.

Characteristic	Number
Age (mean, standard deviation, in years), range	65.8 +/- 8.3, 44-82
Race	
Black White Asian More than one	33 (49.2%) 32 (47.8%) 1 (1.5%) 1 (1.5%)
Ethnicity	
Hispanic Non-Hispanic	15 (22.4%) 52 (77.6%)
Treatment	
Surgery	52 (78%)
*TAH* *“ + BSO* *“ + BSO + PLND* *“ + BSO + PLND + Omentectomy and debulking*	*2* *2* *10* *38*
Initial chemotherapy	65 (97%)
*Carboplatin* *“ + Paclitaxel* *“ + Paclitaxel + Bevacizumab* *“ + Paclitaxel + Herceptin* *“ + Paclitaxel + Pembrolizumab* *“ + Taxotere* *“ + Taxotere + Bevacizumab*	*2* *55* *4* *1* *1* *1* *1*
Radiation therapy	23 (34.3%)
FIGO Stage at diagnosis	
I II III IV	13 (19.4%) 3 (4.5%) 18 (26.9%) 33 (49.2%)
Metastatic disease	33 (49.2%)
Recurrent disease	27 (40.3%)
Mutational status	
*TP53* *PIK3CA* *FBXW7* *ARID1A* *PTEN* *BRCA1* *BRCA2* *CTNNB1* *ERBB2* alterations *AKT1,2,3* *KRAS*	55/58 (94.8%) 14/61 (22.9%) 10/60 (15%) 3/32 (9.4%) 5/57 (8.7%) 2/57 (3.5%) 2/57 (3.5%) 1/58 (1.7%) 12/37 (32.4%) 2/61 (3.3%) 1/61 (1.6%)
Immunohistochemistry	
ER PR PTEN TOP2A TOP01 RRM1 MGMT TS TUBB3	43/65 (66.2%) 24/65 (36.9%) 51/56 (91.1%) 20/22 (90.9%) 14/22 (63.6%) 4/20 (20%) 3/5 (60%) 10/22 (45.5%) 11/22 (50%)

TAH, total hysterectomy; BSO, Bilateral salpingoophorectomy; PLND, pelvic lymph node dissection.

### Molecular aberrations

Molecular profiling was performed on primary tissue at diagnosis on 45/67 patients (67%), on tissue obtained at recurrence in 20/67 patients (30%). In 2/67 patients (3%), molecular profiling was not performed. The most frequently mutated genes identified on NGS were *TP53* (55/58 patients; 94.8%), followed by *PIK3CA* (14/61 patients; 22.9%), and *ERBB2* (12/37 patients; 32%) (**Table 1**). A high tumor mutational burden (TMB) was identified in 14/51 patients (27.5%). On IHC, PTEN was expressed in 51/56 patients (91.1%), ER was expressed in 43/65 patients (66.2%), PR in 24/65 patients (36.9%). None of the patients expressed MMR gene mutation or microsatellite instability (MSI) (**Table 1**).

Fifty-three patients were classified as TCGA copy-number high (79%), six patients as TCGA copy-number low (9%), and 8 patients could not be classified (12%) based on mutational profile.

### Location of metastases and recurrence

Thirty-three patients (49.2%) had evidence of metastases at time of diagnosis (7 on biopsy, 18 on CT, 1 on MRI and 7 on PET/CT), 27 patients (40.3%) had evidence of recurrence during follow-up (3 on biopsy, 13 on CT, and 11 on PET/CT), and 7 patients (10.5%) did not show evidence of metastasis or recurrence during follow-up (**Table 1**).

**Figure 1:**
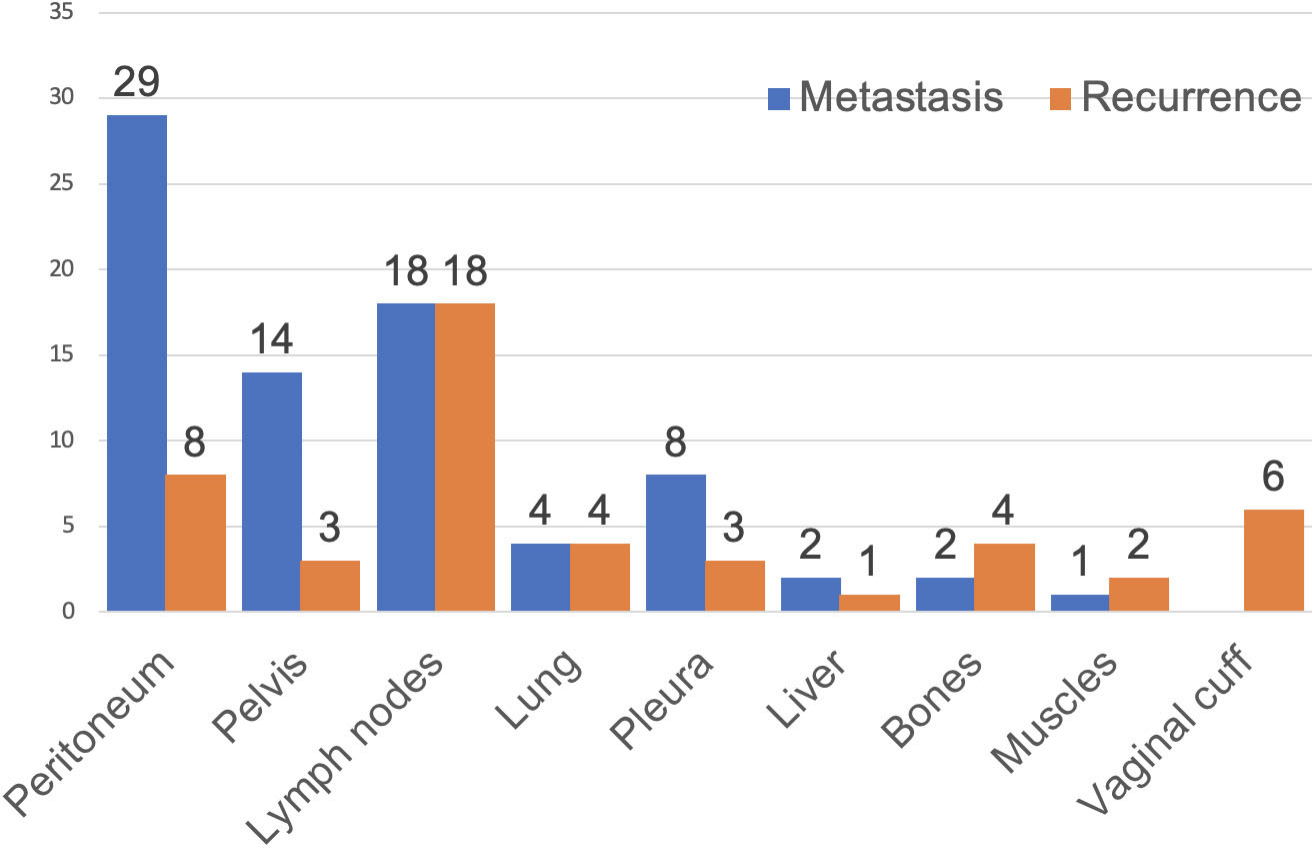
Sites of metastases (blue) and recurrence (orange) in women with USC.

The sites of metastasis and recurrence are summarized in **Figure 1**. The most common sites of metastasis were the peritoneum (29/33, 88%), followed by lymph nodes (18/33, 55%) and in the pelvis (14/33, 42%). The most common site of recurrence was lymph nodes (18/27, 67%), followed by peritoneal implants (8/27, 30%) and vaginal cuff (6/27, 22%).

### Association of mutational status with ethnicity, metastatic and recurrent disease with survival

PR was expressed on IHC more commonly in patients with lymph node metastases (p=0.02), and in non-Hispanic patients (p=0.01). *ERBB2* was more commonly mutated in patients with vaginal cuff recurrence (p=0.02), while *PIK3CA* was more commonly mutated in patients with liver metastases (p=0.048). Peritoneal metastases were significantly more common in Non-Hispanic patients (p=0.04). No other associations between mutations, ethnicity, sites of metastatic and recurrent disease were identified.

**Figure 2:**
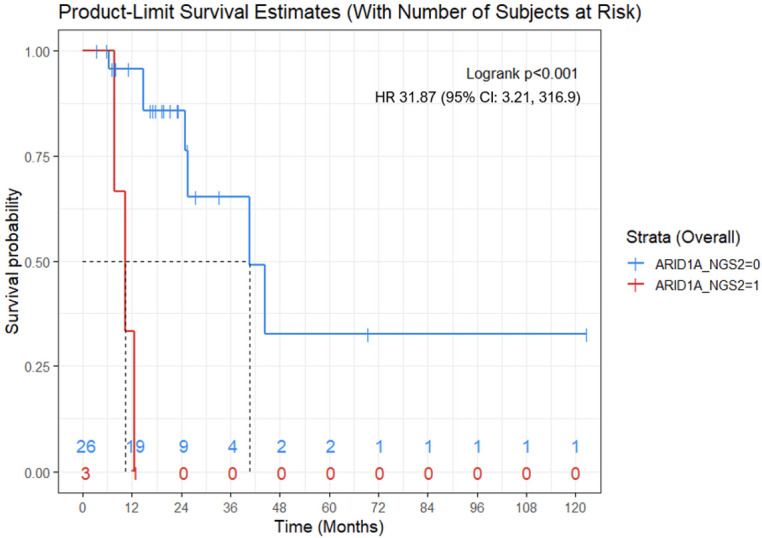
Kaplan-Meier OS curve for women with *ARID1A* mutations (red) versus women without *ARID1A* mutation (blue). ARID1A mutation was associated with shorter OS (mean, 10.2 months vs 60.9 months; Cox proportional hazard model - Hazard Ratio: 31.87; 95% CI: 3.21, 316.9; p<0.001). Numbers at the bottom of the graph refers to the number of women with *ARID1A* mutations (red) versus women without *ARID1A* mutation (blue) at risk at a determinate timepoint.

**Figure 3:**
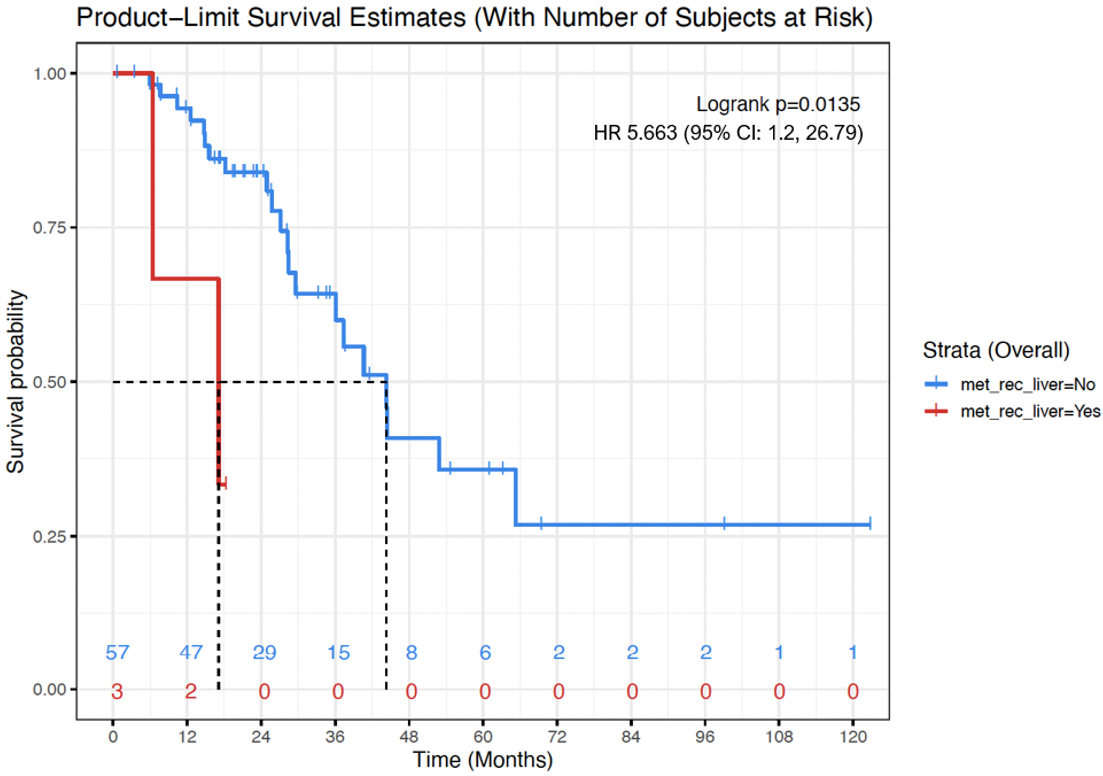
Kaplan-Meier OS curve for women with evidence of liver metastases/recurrence (red) versus women with no evidence or liver metastases or recurrence (blue). Presence of liver metastases/recurrence was associated with shorter OS (mean, 48.8 months vs 58.4 months; Cox proportional hazard model - Hazard Ratio: 5.66; 95% CI: 1.2, 26.79; p=0.01). Numbers at the bottom of the graph refers to the number of women with evidence of liver metastases/recurrence (red) versus women with no evidence or liver metastases or recurrence (blue) at risk at a determinate timepoint.

*ARID1A* mutation was associated with lower OS (mean OS: 10.2 months vs 60.9 months; Hazard Ratio (HR): 31.87; (95% CI: 3.21, 316.9)). Presence of recurrence or metastases to the liver was associated with lower OS (mean OS: 48.8 months vs 58.4 months; HR: 5.66; (95% CI: 1.2, 26.79)) (**Figures 2**, **3**).

### Predictive survival model

A Cox proportional hazards model was selected using a backward stepwise selection process starting with a fully adjusted model. The fully adjusted model included *AKT1,2,3* mutations, presence of recurrence or metastases to the liver, peritoneum, lymph nodes, lungs and a race dummy variable defined as Black or not Black. The predicters in the full model all had log rank p-values<0.20 except for race (p-value=0.59). The Full model was reduced 4 times based on the Wald test statistics for individual variables and the Likelihood Ratio tests (-2 Log Likelihood) resulting in a bivariable Cox model with a total of 60 cases. The final model included presence of recurrence or metastases to the liver, and presence of recurrence or metastases to the peritoneum as predictors (HR: 9.8; 95% CI: 1.85, 52.7; p=0.007 and HR: 2.7; 95% CI: 1.02, 7.1; p=0.04, respectively) (**Table 2**).

**Table 2:** Univariable and multivariable Cox proportional hazard regression analysis of survival in patients with uterine serous carcinoma.

	Univariable Models		Full Model (n=50)		Reduced Model 1 (n=50)		Reduced Model 2 (n=60)		Reduced Model 3 (n=60)		Reduced Model 4 (n=60)	
Variable	HR (95% CI)	P-value	HR (95% CI)	P-value	HR (95% CI)	P-value	HR (95% CI)	P-value	HR (95% CI)	P-value	HR (95% CI)	P-value
Recurrence/Metastases Liver		**0.029**		0.365		0.366		0.100		0.091		**0.007**
	ref	** **	ref		ref		ref		ref		ref	** **
	**5.66 (1.20-26.79)**		3.11 (0.27-36.27)		3.21 (0.26-40.44)		5.27 (0.73-38.22)		5.31 (0.77 -36.75)		**9.88 (1.85-52.75)**	
Recurrence/Metastases Peritoneum		0.098		**0.044**		**0.024**		**0.034**		**0.032**		**0.044**
	ref		ref	** **	ref	** **	ref	** **	ref	** **	ref	** **
	2.14 (0.87-5.25)		**3.53 (1.03-12.03)**		**3.91 (1.19-12.84)**		**2.88 (1.09-7.66)**		**2.89 (1.10-7.62)**		**2.7 (1.03-7.10)**	
Recurrence/Metastases Lung		0.099		0.268		0.325		0.208		0.207		
	ref		ref		ref		ref		ref			
	3.13 (0.81-12.10)		2.92 (0.44-19.42)		2.61 (0.39-17.73)		2.95 (0.55-15.89)		2.95 (0.55-15.83)			
Black		0.587		0.448		0.377		0.973				
	ref		ref		ref		ref					
	1.26 (0.55-2.88)		1.49 (0.53-4.19)		1.59 (0.57-4.44)		1.02 (0.43-2.38)					
*AKT1,2,3*		0.114		0.432		0.481						
	ref		ref		ref							
	5.46 (0.67-44.66)		4.34 (0.11-168.65)	3.65 (0.10-133.79)								
Recurrence/Metastases Lymph Nodes		0.130		0.473								
	ref		ref									
	0.52 (0.22-1.23)		0.68 (0.24-1.94)									
*ARID1A*		**0.003**										
	ref											
	**31.87 (3.21-316.9)**											

HR: Hazard Ratio; CI: Confidence Interval. Bold values relate to the p value <0.05.

## Discussion

With advances in genomic testing and improved understanding of cancer biology, an increasing number of cancer patients undergo mutational testing to identify potential targeted treatment options. As we gain more insight into the impact of the mutational make-up of cancer on prognosis, understanding its associations to known clinicopathologic factors is crucial. Our study cohort was largely comprised of minority Hispanic and Black women that were poorly represented in prior TCGA data and in the PanCancer Atlas (26). Furthermore, 40% of our patients had evidence of recurrence during follow-up while 49% of patients had metastatic disease at diagnosis. This is significantly higher than the TCGA cohort, where 32% presented with recurrence and only 11% had metastatic disease at diagnosis (10).

We showed that *TP53*, *PIK3CA* and *ERBB2* are often mutated in USC, consistent with TGCA data. In our cohort, 79% of cases were classified as copy-number high, consistent with TCGA data, where 77% of cases with serous histology were classified as such (10). In a recent study by Watanabe et al. on 100 EC cases, *TP53* mutations were associated with non-endometrioid histology (12). Several studies have demonstrated that human epidermal growth factor receptor 2 (HER2) a tyrosine kinase increasing cell proliferation encoded by *ERBB2*, is frequently overexpressed in USC (27–29). HER2 overexpression has also been associated with advanced-stage disease, and poorer survival outcomes in USC (25). Few *ARID1A* gene mutations in were identified in our cohort, consistent with the findings of the PanCancer Atlas, where *ARID1A* gene mutations were identified in 13% of cases (26, 30). Mutations in *ARID1A* in EC have been associated with promoting tumor invasion and metastasis, which may shed light on the significant mortality among women diagnosed with USCs (30).

In our cohort, ER and PTEN are expressed in the majority of USC on IHC. A study on 1054 women with EC showed that ER, though more common in type I EC, was expressed in 72% of type II EC (31). A study on 56 high grade EC showed that majority of USC were positive for PTEN on IHC (32). A study on 221 women with EC, showed that loss of PTEN expression was associated with endometrioid histology and favorable survival (33).

In our study, *FBXW7* was mutated in 15% of USC. No association between *FBXW7* mutations, ethnicity and race was identified in our study. *FBXW7* was mutated in 29 out of 109 (27%) USC included in the PanCancer Atlas. In a study on 66 USC, *FBXW7* mutations were identified in 18.2% of cases (11). On the study by Watanabe et al., *FBWX7* mutations were associated with late-stage, vascular invasion, and lymph node metastasis (12).

In our cohort, USC most commonly metastasized to lymph nodes, peritoneum and pelvic organs, while recurrences were mostly nodal, peritoneal and at the vaginal cuff. These findings are similar to two prior studies: a study on 841 EC, which showed that the most common sites of recurrence of USC was the abdomen, including ascites, followed by the vaginal cuff and a study on 50 USC, which showed that the most common sites of metastases of USC were the lymph nodes, pelvic organs and peritoneum/omentum (13, 14).

In our study, PR expression was associated with non-Hispanic ethnicity and nodal metastases. A study on 99 endometrioid EC showed that nodal metastases correlated with negative PR status on IHC (34). This discrepancy may be related to the different histologies. The association between non-Hispanic ethnicity and PR expression is unclear.

We found that *ERBB2* alterations were more common in patients with vaginal cuff recurrence and *PIK3CA* mutation was more common in patients with liver metastases. *ERBB2* amplifications are associated with higher stage, chemoresistance, and lower survival, especially in Black patients (35, 36). Trastuzumab, a HER2/neu receptor inhibitor monoclonal antibody, demonstrated increased progression free survival when added to carboplatin paclitaxel, in patients with USC and ERBB2 overexpression (37). This highlights the significance of somatic tumor testing either at diagnosis or at recurrence to aid in prioritizing treatment options.

Women with presence of recurrence or metastases to the liver and *ARID1A* mutations had lower OS. A study on 86 recurrent EC, showed that recurrence to the liver was associated with lower OS, with an HR of 10 [3.72–26.81 95% CI] (14). A metanalysis on the prognostic significance of ARID1A in endometrium-related gynecological cancers showed that negative ARID1A expression predicted shorter progression free survival (38). *ARID1A* mutations affects multiple pathways, and may mediate resistance to platinum chemotherapy, possibly explaining the lower OS observed in our study (39). Therapies targeting the pathways affected by *ARID1A* mutations, such as poly(ADP-ribose) polymerase inhibitors, immune checkpoint inhibitors and mTOR inhibitors, have shown activity in preclinical models and in patient, and could be implemented in patients with *ARID1A*-mutated USC (40, 41).

Finally, on multivariable Cox proportional hazards regression analysis, the presence of recurrence or metastases to the liver and/or the peritoneum were independently associated with shorter OS regardless of their mutational status, race, ethnicity or other sites of recurrence or metastases. Various studies attempted to build predictive survival models for USC, including a study by Chen et al. on 110 women with USC which showed that a combination of mutated genes, a 4-gene signature, was predictive of OS (42). Differently from our study, their model did not include sites of metastases or survival as potential predictors. Knowledge of the associations between survival data and sites of recurrence or metastases of USC is valuable: it allows for a more accurate risk stratification and helps the oncologists and radiologists to potentially formulate more appropriate follow-up strategies (43).

Some limitations of this study should be noted. This is a retrospective study performed at a single institution with inherent selection bias. The relatively small patient cohort and the widely variable follow-up period may have limited the power in detection of some of the associations between mutations and patterns of metastases and recurrence, as well as the prognostic values of mutations. Furthermore, our sample included only patients with USC selected from a tertiary cancer center and may not be representative of patients treated at a community hospital. We grouped different genetic aberrations involving the same gene under that gene to facilitate analysis until additional data are available. This methodology has also been previously utilized and reported (11, 21).

One of the significant strengths of this study is the cohort of Hispanic and Black women diagnosed with USC with genomic testing. Our study showed that mutational status of USC had implications on pattern of metastases and survival, and that sites of recurrence and metastases influence survival. These findings should be assessed in larger studies, to confirm our findings and may be valuable for future trial design.

## Data availability statement

The raw data supporting the conclusions of this article will be made available by the authors, without undue reservation.

## Ethics statement

The studies involving human participants were reviewed and approved by University of Miami. Written informed consent for participation was not required for this study in accordance with the national legislation and the institutional requirements.

## Author contributions

MH: Supervision, ideation, and draft editing. SW: statistical analysis and draft editing. FA: ideation, statistical analysis, draft writing, and editing. CL: data collection and draft editing. NG: data collection MM: data collection and draft editing. All authors contributed to the article and approved the submitted version.

## References

[1] US Surveillance, Epidemiology, and End Results (SEER). Cancer stat facts - uterine cancer (2022). Available at: https://www.seer.cancer.gov/statfacts/html/corp.html (Accessed January 5, 2022).

[2] Siegel RL Miller KD Fuchs HE Jemal A. Cancer statistics, 2021. CA: A Cancer J Clin (2021) 71(1):7–33. doi: 10.3322/caac.21654 33433946

[3] Morice P Leary A Creutzberg C Abu-Rustum N Darai E. Endometrial cancer. Lancet (2016) 387(10023):1094–108. doi: 10.1016/S0140-6736(15)00130-0 26354523

[4] Lu KH Broaddus RR. Endometrial cancer. New Engl J Med (2020) 383(21):2053–64. doi: 10.1056/NEJMra1514010 33207095

[5] del Carmen MG Birrer M Schorge JO. Uterine papillary serous cancer: a review of the literature. Gynecol Oncol (2012) 127(3):651–61. doi: 10.1016/j.ygyno.2012.09.012 23000148

[6] Brooks RA Fleming GF Lastra RR Lee NK Moroney JW Son CH. Current recommendations and recent progress in endometrial cancer. CA: A Cancer J Clin (2019) 69(4):258–79. doi: 10.3322/caac.21561 31074865

[7] Setiawan VW Yang HP Pike MC McCann SE Yu H Xiang YB. Type I and II endometrial cancers: have they different risk factors? J Clin Oncol: Off J Am Soc Clin Oncol (2013) 31(20):2607–18. doi: 10.1200/JCO.2012.48.2596 PMC3699726 23733771

[8] Luna C Balcacer P Castillo P Huang M Alessandrino F. Endometrial cancer from early to advanced-stage disease: an update for radiologists. Abdominal Radiol (New York) (2021) 46(11):5325–36. doi: 10.1007/s00261-021-03220-7 34297164

[9] Arciuolo D Travaglino A Raffone A Raimondo D Santoro A Russo D. TCGA molecular prognostic groups of endometrial carcinoma: current knowledge and future perspectives. Int J Mol Sci (2022) 23(19):11684. doi: 10.3390/ijms231911684 36232987 PMC9569906

[10] Levine DA. Integrated genomic characterization of endometrial carcinoma. Nature (2013) 497(7447):67–73. doi: 10.1038/nature12113 23636398 PMC3704730

[11] Kuhn E Wu RC Guan B Wu G Zhang J Wang Y. Identification of molecular pathway aberrations in uterine serous carcinoma by genome-wide analyses. J Natl Cancer Inst (2012) 104(19):1503–13. doi: 10.1093/jnci/djs345 PMC3692380 22923510

[12] Watanabe T Nanamiya H Kojima M Nomura S Furukawa S Soeda S. Clinical relevance of oncogenic driver mutations identified in endometrial carcinoma. Trans Oncol (2021) 14(3):101010. doi: 10.1016/j.tranon.2021.101010 PMC7810788 33450701

[13] Goff BA Kato D Schmidt RA Ek M Ferry JA Muntz HG. Uterine papillary serous carcinoma: patterns of metastatic spread. Gynecol Oncol (1994) 54(3):264–8. doi: 10.1006/gyno.1994.1208 8088602

[14] Rosenberg P Blom R Högberg T Simonsen E. Death rate and recurrence pattern among 841 clinical stage I endometrial cancer patients with special reference to uterine papillary serous carcinoma. Gynecol Oncol (1993) 51(3):311–5. doi: 10.1006/gyno.1993.1296 8112638

[15] Sohaib SA Houghton SL Meroni R Rockall AG Blake P Reznek RH. Recurrent endometrial cancer: patterns of recurrent disease and assessment of prognosis. Clin Radiol (2007) 62(1):28–34. doi: 10.1016/j.crad.2006.06.015 17145260

[16] Sorbe B Juresta C Ahlin C. Natural history of recurrences in endometrial carcinoma. Oncol Lett (2014) 8(4):1800–6. doi: 10.3892/ol.2014.2362 PMC4156268 25202413

[17] Creutzberg CL van Putten WL Koper PC Lybeert ML Jobsen JJ Wárlám-Rodenhuis CC. Surgery and postoperative radiotherapy versus surgery alone for patients with stage-1 endometrial carcinoma: multicentre randomised trial. PORTEC study group. post operative radiation therapy in endometrial carcinoma. Lancet (2000) 355(9213):1404–11. doi: 10.1016/S0140-6736(00)02139-5 10791524

[18] Legge F Restaino S Leone L Carone V Ronsini C Di Fiore GLM. Clinical outcome of recurrent endometrial cancer: analysis of post-relapse survival by pattern of recurrence and secondary treatment. Int J Gynecol Cancer (2019) 30(2):193–200. doi: 10.1136/ijgc-2019-000822 31792085

[19] Ferriss JS Erickson BK Shih IM Fader AN. Uterine serous carcinoma: key advances and novel treatment approaches. Int J Gynecol Cancer (2021) 31(8):1165–74. doi: 10.1136/ijgc-2021-002753 34210768

[20] Uygur MC Yaman I Kutluay L Altuğ U Erol D. The relation between p53 overexpression and lymph node metastases in clinical stage t2 and t3a transitional cell bladder carcinoma. J Exp Clin Cancer Res (1999) 18(3):391–5. 10606186

[21] Park DS Lee YT Lee JM. Prediction of lymph node metastasis based on p53 and nm23-H1 expression in muscle invasive grade III transitional cell carcinoma of bladder. Adv Exp Med Biol (2003) 539(Pt A):67–85. doi: 10.1007/978-1-4419-8889-8_6 15088897

[22] Alessandrino F Williams K Nassar AH Silverman SG Sonpavde G Shinagare AB. Muscle-invasive urothelial cancer: association of mutational status with metastatic pattern and survival. Radiology (2020) 295(3):572–80. doi: 10.1148/radiol.2020191770 32228295

[23] Eisenhauer EA Therasse P Bogaerts J Schwartz LH Sargent D Ford R. New response evaluation criteria in solid tumours: revised RECIST guideline (version 1.1). Eur J Cancer (2009) 45(2):228–47. doi: 10.1016/j.ejca.2008.10.026 19097774

[24] U.S. Food and Drug Administration. FoundationOne CDx– P170019/S014 technical information. Available at: https://www.accessdata.fda.gov/cdrh_docs/pdf17/P170019S006C.pdf (Accessed December 20, 2022).

[25] Caris Life Sciences. Comprehensive molecular profiling. Available at: https://www.carislifesciences.com/products-and-services/molecular-profiling/ (Accessed December 20, 2022).

[26] Uterine corpus endometrial carcinoma (TCGA, PanCancer atlas). Available at: https://www.cbioportal.org/study/summary?id=ucec_tcga_pan_can_atlas_2018 (Accessed July 30, 2022).

[27] Singh P Smith CL Cheetham G Dodd TJ Davy MLJ. Serous carcinoma of the uterus–determination of HER-2/neu status using immunohistochemistry, chromogenic *in situ* hybridization, and quantitative polymerase chain reaction techniques: its significance and clinical correlation. Int J Gynecol Cancer (2008) 18(6):1344–51. doi: 10.1111/j.1525-1438.2007.01181.x 18248390

[28] Slomovitz BM Broaddus RR Burke TW Sneige N Soliman PT Wu W. Her-2/neu overexpression and amplification in uterine papillary serous carcinoma. J Clin Oncol (2004) 22(15):3126–32. doi: 10.1200/JCO.2004.11.154 15284264

[29] Villella JA Cohen S Smith DH Hibshoosh H Hershman D. HER-2/neu overexpression in uterine papillary serous cancers and its possible therapeutic implications. Int J Gynecol Cancer (2006) 16(5):1897–902. doi: 10.1111/j.1525-1438.2006.00664.x 17009989

[30] Suryo Rahmanto Y Shen W Shi X Chen X Yu Y Yu ZC. Inactivation of Arid1a in the endometrium is associated with endometrioid tumorigenesis through transcriptional reprogramming. Nat Commun (2020) 11(1):2717. doi: 10.1038/s41467-020-16416-0 PMC7264300 32483112

[31] Shen F Gao Y Ding J Chen Q. Is the positivity of estrogen receptor or progesterone receptor different between type 1 and type 2 endometrial cancer? Oncotarget (2016) 8(1):506–11. doi: 10.18632/oncotarget.13471 PMC5352172 27888807

[32] Gilks CB Oliva E Soslow RA. Poor interobserver reproducibility in the diagnosis of high-grade endometrial carcinoma. Am J Surg Pathol (2013) 37(6):874–81. doi: 10.1097/PAS.0b013e31827f576a 23629444

[33] Akiyama-Abe A Minaguchi T Nakamura Y Michikami H Shikama A Nakao S. Loss of PTEN expression is an independent predictor of favourable survival in endometrial carcinomas. Br J Cancer (2013) 109(6):1703–10. doi: 10.1038/bjc.2013.455 PMC3776978 23949151

[34] Iwai. K Fukuda. K Hachisuga. T Mori. M Uchiyama. M Iwasaka. T. Prognostic significance of progesterone receptor immunohistochemistry for lymph node metastases in endometrial carcinoma. Gynecol Oncol (1999) 72(3):351–9. doi: 10.1006/gyno.1998.5286 10053107

[35] Santin AD Bellone S Siegel ER Palmieri M Thomas M Cannon MJ. Racial differences in the overexpression of epidermal growth factor type II receptor (HER2/neu): a major prognostic indicator in uterine serous papillary cancer. Am J Obstet Gynecol (2005) 192(3):813–8. doi: 10.1016/j.ajog.2004.10.605 15746676

[36] Santin AD Bellone S Van Stedum S Bushen W Palmieri M Siegel ER. Amplification of c-erbB2 oncogene. Cancer (2005) 104(7):1391–7. doi: 10.1002/cncr.21308 16116605

[37] Fader AN Roque DM Siegel E Buza N Hui P Abdelghany O. Randomized phase II trial of carboplatin-paclitaxel versus carboplatin-Paclitaxel-Trastuzumab in uterine serous carcinomas that overexpress human epidermal growth factor receptor 2/neu. J Clin Oncol: Off J Am Soc Clin Oncol (2018) 36(20):2044–51. doi: 10.1200/JCO.2017.76.5966 29584549

[38] Liu G Xu P Fu Z Hua X Liu X Li W. Prognostic and clinicopathological significance of ARID1A in endometrium-related gynecological cancers: a meta-analysis. J Cell Biochem (2017) 118(12):4517–25. doi: 10.1002/jcb.26109 28466574

[39] Mullen J Kato S Sicklick JK Kurzrock R. Targeting ARID1A mutations in cancer. Cancer Treat Rev (2021) 100:102287. doi: 10.1016/j.ctrv.2021.102287 34619527

[40] Samartzis EP Noske A Dedes KJ Fink D Imesch P. ARID1A mutations and PI3K/AKT pathway alterations in endometriosis and endometriosis-associated ovarian carcinomas. Int J Mol Sci (2013) 14(9):18824–49. doi: 10.3390/ijms140918824 PMC3794809 24036443

[41] Okamura R Kato S Lee S Jimenez RE Sicklick JK Kurzrock R. ARID1A alterations function as a biomarker for longer progression-free survival after anti-PD-1/PD-L1 immunotherapy. J Immunother Cancer (2020) 8(1):e000438. doi: 10.1136/jitc-2019-000438 32111729 PMC7057434

[42] Chen H Li L Qin P Xiong H Chen R Zhang M. A 4-gene signature predicts prognosis of uterine serous carcinoma. BMC Cancer (2021) 21(1). doi: 10.1186/s12885-021-07834-4 PMC7881619 33579221

[43] Shinagare AB Ip IK Lacson R Ramaiya NH George S Khorasani R. Gastrointestinal stromal tumor: optimizing the use of cross-sectional chest imaging during follow-up. Radiology (2015) 274(2):395–404. doi: 10.1148/radiol.14132456 25203129

